# Methoxyfenozide tolerance in *Chrysoperla carnea*: Inheritance, dominance and preliminary detoxification mechanisms

**DOI:** 10.1371/journal.pone.0265304

**Published:** 2022-03-22

**Authors:** Muhammad Mudassir Mansoor, Sarfraz Ali Shad

**Affiliations:** 1 Fatima Sugar Research & Development Centre, Fatima Sugar Mills Ltd, Muzaffargarh, Punjab, Pakistan; 2 Department of Entomology, Faculty of Agricultural Sciences and Technology, Bahauddin Zakariya University, Multan, Punjab, Pakistan; Ghent University, BELGIUM

## Abstract

Lacewings exist in insecticide-dominant cropping systems. They are prime biological control agents due to outstanding ability of insecticide resistance development. This study examines occurrence of methoxyfenozide resistance and its subsequent effects on cross-resistance to other insecticides, inheritance and mechanism of resistance in *C*. *carnea*. Methoxy-SEL strain of *C*. *carnea* selected for 15 generations developed 3531.67-fold resistance to methoxyfenozide. Overlapping fiducial limits of LC_50_s of F_1_ and F_1’_ (reciprocal crosses) suggested an autosomal and incompletely dominant mode of inheritance. Resistance to methoxyfenozide was polygenic and its realized heritability value was high (h^2^ = 0.62). Both PBO and DEF significantly changed LC_50_s indicating cytochrome P450-dependent monooxygenases and esterases detoxifying the resistance in Methoxy-SEL strain. Resistance to all tested insecticide was unstable but decrease rate was very negligible. These results have implications forpreservation of biological control and effective use in insecticide-dominant cropping systems.

## Introduction

A key element of pest management strategies in agro-ecosystems is to develop basic understanding of the impact on non-target natural enemies [1]. Insecticides prevail as a dominant tool for pest management regardless of having potential consequences on non-target arthropods [2]. Biological control is one of the constituents of different Integrated Pest Management (IPM) programs in multiple cropping situations [3]. Although, developing compatibility between insecticides and natural enemies has been portrayed as debatable and complicated issue [4], but both are still being used in agriculture.

*Chrysoperla carnea* (Stephens) (Neuroptera:Chrsopidae) is an effective voracious predator. It is widely used against several insect pests including whiteflies, aphids, jassids [5], thrips, mites, mealybugs and lepidoptera eggs. Due to its effectiveness, this predator is commercially available worldwide making it a cosmopolitan species. It has developed remarkable resistance to several groups of insecticides including conventional, pyrethroids [5, 6], novel mode of action [7–9] and insect growth regulators (IGRs) [10–12].

To make an IPM program successful, it is important to use selective insecticides in tandem with natural enemies and other management tactics. This may deliver comprehensive management of target pests better than either approach singly [13]. Selectivity of insecticides with respect to biological control agents can be evaluated in the field and laboratory, but most studies have been done in controlled laboratory conditions. This is due to the probability of uncontainable biotic and abiotic pressures in field studies [14].

Characterization of different aspects of insecticide resistance is essential to recognize the phenomenon of resistance development. For this purpose, studying insecticide resistance, genetics, mechanism, and other relevant features is important [15, 16]. It includes aknowledgebase for designing effective IPM strategy including natural enemies while eliminating target pests. Studies reporting insecticide resistance, cross-resistance, inheritance, realized heritability mechanism and stability in *C*. *carnea* have been conducted [5, 7–10, 17]. However, there is no report of methoxyfenozide resistance and its characterization for this natural enemy.

Methoxyfenozide is an insect growth regulator (IGR) with ecdysone receptor agonist action [18]. This IGR is quite selective in action, posses very low acute toxicity on mammals, harmful to target pest species [19], and compatible with natural enemies [20]. Recently, several resistance aspectsof this IGR havebeen documented from several countries such as selection [21], resistance risk assessment and baseline monitoring [22], toxicity and kinetics in *Spodoptera exigua* (Hübner) [23], mechanism and stability in *Spodoptera litura* (Fabricius) [24], cross-resistance and fitness costs [25] and genetics in *Musca domestica* L. [26]. Information about the effects of methoxyfenozide on natural enemies is very limited. Hewa-Kapuge et al., 2003 [27] studied the effects of this IGR on an egg parasitoid *Trichogramma barassicae* Bez. (Hymenoptera: Trichogrammatidae) and concluded that it is potentially suitable to control pests in the presence of this natural enemy. As *C*. *carnea* is an admired and dominant predator existing in multiple cropping systems, it is important to study methoxyfenozide resistance and its aspects on this natural enemy.

## Materials and methods

### *C*. *carnea* collection and rearing

About 300 adults of *C*. *carnea* were collected with a ventilated plastic vial (15 x 45 mm) from cotton, sugarcane and vegetable fields in District Muzaffargarh ((30.0703° N, 71.1933° E) in early spring. Verbal consent of local growers was obtained before collections from different fields so no legal permits were necessary in this regard. These adults were shifted to the Biological Control Laboratory, Fatima Sugar Research & Development Centre, District Muzaffargarh, in plastic cages (23 x 38 x 38 cm) and reared on an artificial diet mixture of water, honey and yeast (4:2:1 ratio) [6, 7]. Black glossy papers were instantly lined with ceiling of rearing cares for egg laying and replaced within 24 hours. At least 5–10 eggs removed from this black glossy paper by a sharp knife were placed inside transparent gelatin capsule (500 mg). A culture of Angoumois grain moth, *Sitotroga cerealella* Oliver was taken from IPM Station PARC, Faculty of Agricultural Sciences and Technology Bahauddin Zakariya University, Multan in 2007 and reared without any insecticide exposure [17]. Eggs of *S*. *cerealella* were placed in the transparent capsule to feed the *C*. *carnea* larvae. Temperature, relative humidity and photoperiod were maintained as previously reported in recent publications [8, 9].

### Insecticides

Formulated insecticides used for bioassays include methoxyfenozide (Runner^®^ 240SC, Arysta Life Sciences, Pakistan) 100–800 μg ml^-1^, acetamiprid (Mospilon ^®^ 20 WP, Dow Agro-Sciences) 250–2000 μg ml^-1^, profenofos (Curacron^®^ 500 EC, Syngenta, Pakistan) 25–200 μg ml^-1^ and lambda-cyhalothrin, (Karate^®^ 2.5 EC, Syngenta, Pakistan) 50–400 μg ml^-1^. For synergism tests, Piperonyl butoxide (PBO; Sigma Ltd, UK), inhibitors of cytochrome P450 monooxygenases (microsomal oxidases) & esterases, (DEF; Sigma Ltd, UK), an another esterase specific inhibitor, S,S,S-tributylphosphorotrithioate were used.

### Concentration-response bioassays

Topical bioassays were performed to evaluate toxicity of tested insecticides on 1^st^ instar larvae of *C*. *carnea* (Field). A droplet of 0.5 μl was applied from 1-ml glass syringe of Micro-applicator (Burkard Manufacturing Co. Ltd., Hertfordshire, England) directly on thorax of larvae [6]. Four serial concentrations of each tested insecticide were made and each concentration was replicated four times. There were 20 larvae in each replication making a total of 320 larvae treated perinsecticide. However, only 30 larvae of *C*. *carnea* were treated with distilled water as control [28].

A susceptible strain of *C*. *carnea* (Susceptible) which was obtained in 2007 and reared at Biological Control Laboratory, Fatima Sugar Research & Development Centre, District Muzaffargarh was also used in bioassays. Larvae exposed to distilled water or the treatments were kept in transparent capsules with eggs of *S*. *cerealella* till pupation [29].

### Selection with methoxyfenozide

Larvae of *C*. *carnea* (Field) were grouped into two sub-groups. At least 100 adults were included in each group. One sub-group (UNSEL) was reared without any insecticide treatment while second sub-group (Methoxy-SEL) was treated with varying levels (800 to 12800 μg ml^-1^) of methoxyfenozide from G1 to G15 [9, 10]. Larvae were treated as reported in concentration-response bioassay section.

### Genetic crosses

At least 30 male adults from Methoxy-SEL and 30 female adults from Susceptible strain were crossed to obtain F_1_. Similarly, 30 female adults from Methoxy-SEL and 30 male adults from Susceptible strain were mated to get F_1’_. Another strain, F_2_ was developed by crossing 30 males and females of F_1_ strain. A Backcross (BC_1_) was also developed using 30 females from F_1_ strain and 30 males from Susceptible strain.

#### Degree of dominance (D_LC_)

D_LC_ of methoxyfenozide resistance was estimated with the following formula. Resistance was assumed completely dominant (if D_LC_ = 1) and completely recessive (if D_LC_ = 0) [30].

$$D_{LC}=\left(\text{log}\;{\text{LC}}_{\text{RS}}-\text{log}\;{\text{LC}}_{\text{S}}\right)÷\left({\text{log}\;\text{LC}}_{\text{R}}-\text{log}\;{\text{LC}}_{\text{S}}\right).$$

Where Log LC_R_ = LC_50_ of Methoxy-SEL, LC_RS_ = LC_50_ of F_1_ and LC_S_ = LC_50_ of Susceptible strains [31].

Effective dominance (D_ML_) of resistance to methoxyfenozide was estimated as

$${\text{D}}_{\text{ML}}=\left({\text{MT}}_{\text{RS}}-{\text{MT}}_{\text{SS}}\right)÷\left({\text{MT}}_{\text{RR}}-{\text{MT}}_{\text{SS}}\right).$$

Where MT_RS_ (F_1_), MT_RR_ (Methoxy-SEL) and MT_SS_ (Susceptible) shows percent mortality on any single tested dose of insecticide. Resistance to methoxyfenozide was completely dominant (if D_ML_ = 1) and completely recessive (if D_ML_ = 0) [32].

#### Calculation of gene frequency

Monogenic resistance hypothesis was tested using Goodness of fit (Chi-square) test [33]. Equation for testing null hypothesis of monogenic resistance is given below.

$$\chi^{2}={\left(\text{F}-\text{pn}\right)}^{2}÷\text{pqn}.$$

Where F is mortality in BC_1_, p is expected mortality, n is total larvae exposed to any dose and q is 1-p. Null hypothesis of monogenic resistance get rejected if 50% expected and observed mortalities show significant difference (p > 0.05).

Gene frequency responsible for methoxyfenozide resistance can be calculated as

$$\eta E={\left(X_{RR}-X_{SS}\right)}^{2}÷\left(8\sigma^{2}S\right).$$

Where X_RR_ = Log of LC_50_ of Methoxy-SEL and X_SS_ = Log of LC_50_ of Susceptible strain [34].

The σ^2^S was estimated as follow:

$$\sigma^{2}S=\sigma^{2}B_{1}-\left[\sigma^{2}F_{1}+0.5\;\sigma^{2}X_{SS}+\sigma^{2}X_{RR}\right]$$

Where σ^2^B_1_, σ^2^F_1_, σ^2^ X_SS_, and σ^2^ X_RR_ are variances of BC_1_, F_1_, Susceptible and Methoxy-SEL strains.

### Realized heritability (*h*^*2*^)

Realized heritability of methoxyfenozide resistance was calculated as follows.


$$h^{2}=\text{Response}\;\text{to}\;\text{selection}\;\left(R\right)\;/\;\text{Selection}\;\text{differential}\;\left(S\right).$$

[35]. Response to selection was calculated as

$$R=\left[\text{Log}\;\text{final}\;{\text{LC}}_{50}\;\text{of}\;\text{Methoxy}-\text{SEL}-\text{Log}\;\text{initial}\;{\text{LC}}_{50}\;\text{of}\;\text{Field}\;\text{Pop}\right]/n,$$

Here, *n* shows total number of generations exposed to methoxyfenozide.

Selection differential (*S*) was calculated with given formula:

$$S=\text{Intensity}\;\text{of}\;\text{selection}\;\left(i\right)\times \text{Phenotypic}\;\text{standard}\;\text{deviation}\;\left(\sigma p\right).$$

Intensity of selection was as,

i = 1.583 − 0.0193336p + 0.0000428p^2^ + 3.65194 / p,

Where *p* is average survival of Methoxy-SEL strain.

Phenotypic standard deviation (σp) was determined as

$$\sigma \text{p}={\left[1/2\left(\text{final}\;\text{slope}+\text{initial}\;\text{slope}\right)\right]}^{-1}.$$

### Biochemical mechanism

PBO and DEF (5 mg ml^-1^) were diluted in acetone and mixed with insecticide concentrations. Experiments to determine the resistance mechanism were conducted as previously reported by Mansoor et al., 2017 [7].

### Stability and decrease rate of resistance

The sub-group (UNSEL) left unexposed to any insecticide was used for this study. Resistance stability and its decrease rate (DR) to tested insecticides was measured using given formula [36].

$$\text{DR}=\left[\text{log}\;\left({\text{final}\;\text{LC}}_{50}\right)-\text{log}\;\left({\text{initial}\;\text{LC}}_{50}\right)\right]\;÷\text{n}$$

Total number of *C*. *carnea* generations required for a 10-fold decrease in resistance to methoxyfenozide and other tested insecticides was also calculated as follows.

GR=1/R

where *R* is response to selection.

### Data collection and statistical analysis

Treated larvae were tapped softly by a needle-like brush after 72 hours of bioassays. These were considered dead if there was no movement. Mortality data was corrected [37] if necessary. Mortality data was further analyzed with Probit Analysis [38]. These analyses produced Median Lethal Concentration (LC_50_), 95% Fiducial Limits (FLs), standard errors and slope values. LC_50_s were regarded significantly different if FLs had no overlapping [39, 40].

## Results

### Toxicity of various insecticides to Susceptible, field, UNSEL and Methoxy-SEL strains

**Fig 1:**
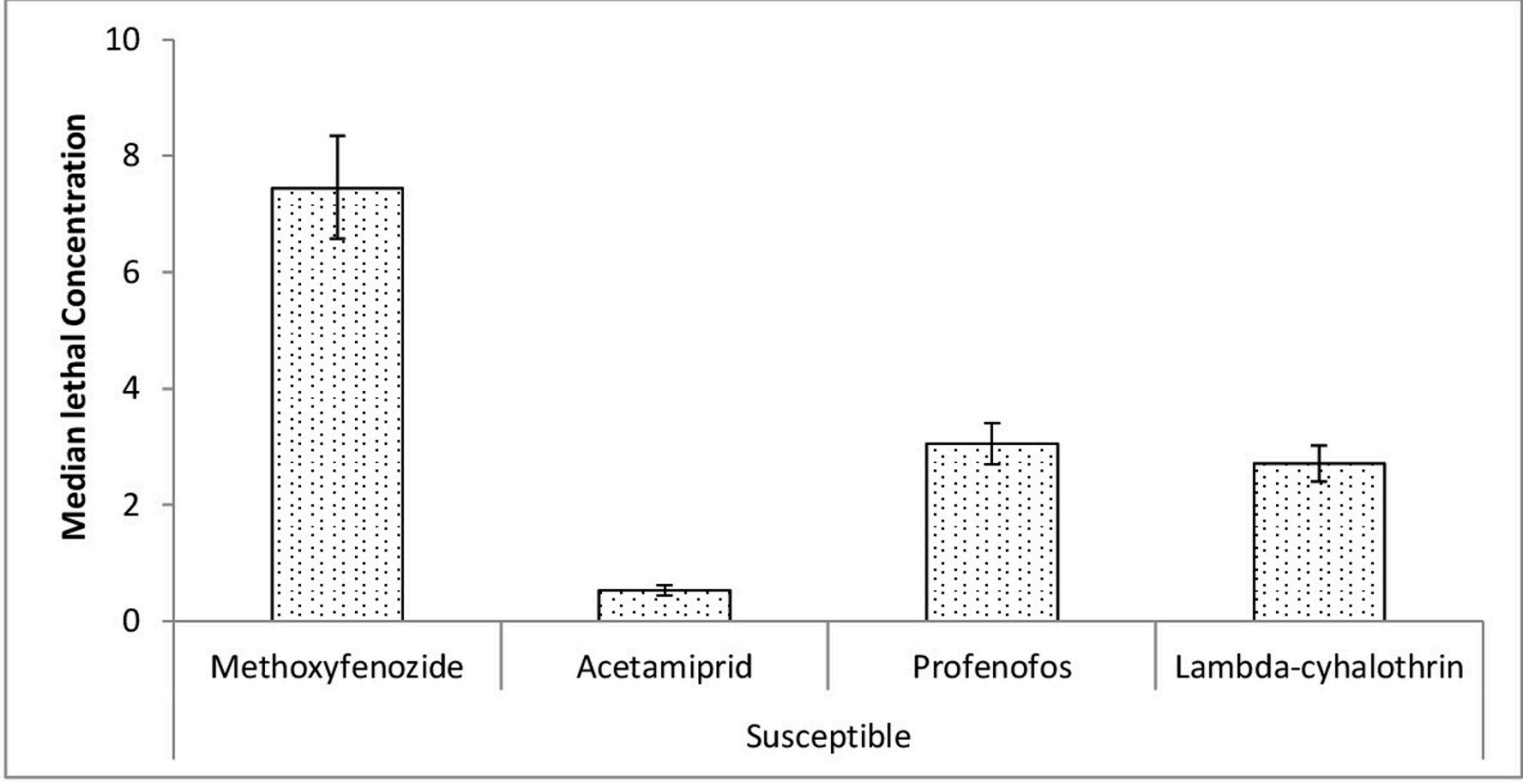
Toxicity of various insecticides to susceptible strain of *Chrysoperla carnea*. Error Bars show fiducial limits (FLs) of LC_50_s.

Methoxyfenozide was significantly less toxic to Susceptible strain followed by profenofos and lambda-cyhalothrin (95% FLs didn’t overlap). Toxicity of profenofos and lambda-cyhalothrin was significantly similar (95% FLs overlapping). Acetamiprid was the most toxic insecticide than all other tested insecticides (95% FLs didn’t overlap) (Fig 1).

**Fig 2:**
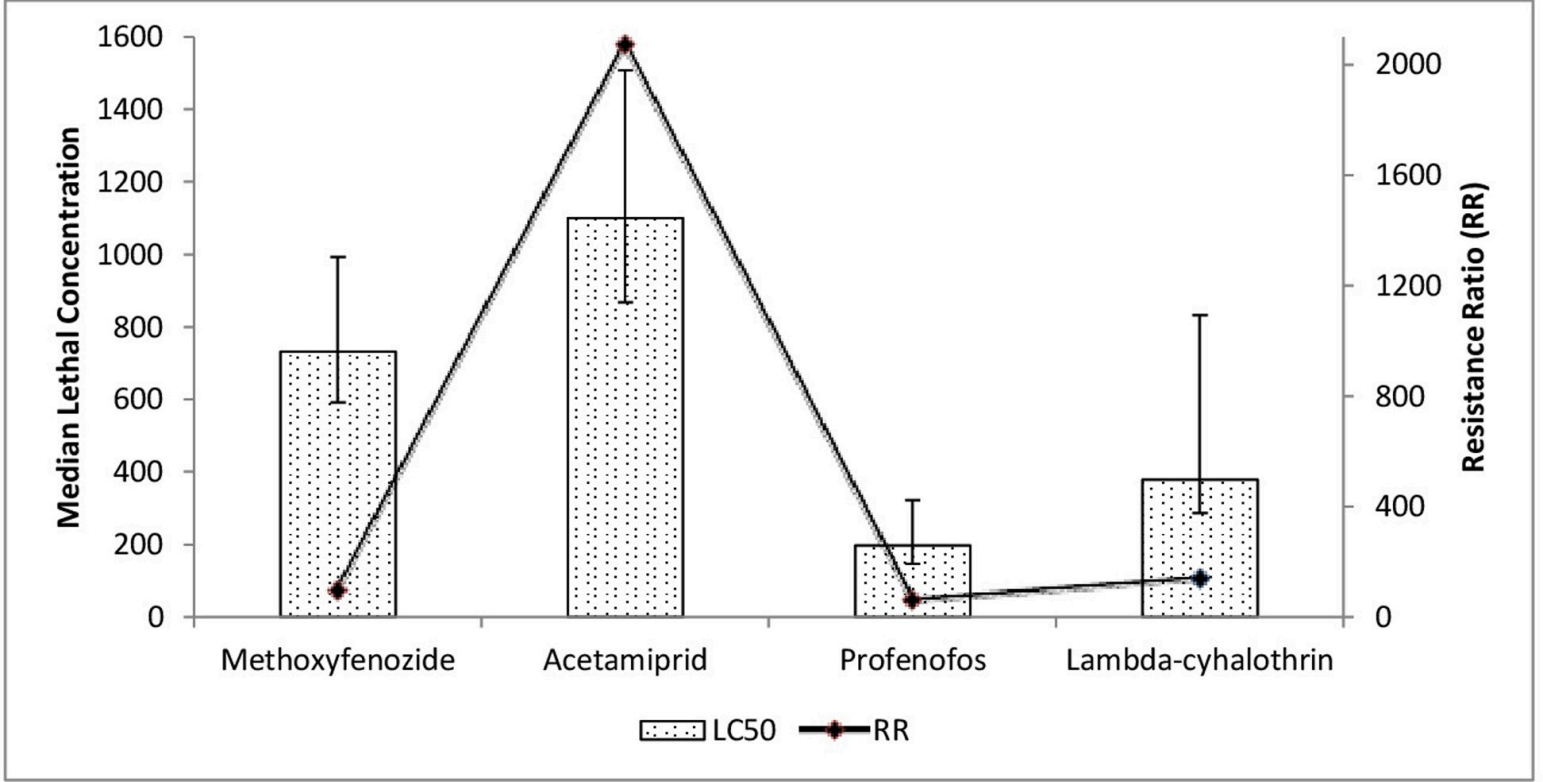
Toxicity of various insecticides to field strain (G1) of *Chrysoperla carnea* and resistance ratios. Error Bars show fiducial limits (FLs) of LC_50_s.

For field strain, methoxyfenozide was significantly less toxic than profenofos (95% FLs didn’t overlap). However, it was relatively similar to that of acetamiprid and lambda-cyhalothrin (95% FLs overlapping). Profenofos and lambda-cyhalothrin were the most toxic insecticides. Toxicity to acetamiprid was significantly lower than profenofos and lambda-cyhalothrin (95% FLs didn’t overlap) (Fig 2).

The toxicity of methoxyfenozide was significantly low compared to other tested insecticides (95% FLs didn’t overlap). Acetamiprid was less toxic than lambda-cyhalothrin (95% FLs didn’t overlap). However, toxicity of profenofos and lambda-cyhalothrin was significantly high and similar (95% FLs overlapping) (Table 1).

**Table 1:** Toxicity of various insecticides to Methox-SEL population of *Chrysoperla carnea*.

Strain	Insecticide	LC_50_ (95% FLs) μg ml^-1^	Fit of probit line				N^a^	RR^b^	RR^c^
			Slope (±SE)	χ2	Df	*P*			
Methox-SEL (G15)	Methoxyfenozide	26275.66(16686.46–67071.95)	1.56(0.30)	0.30	3	0.96	350	3531.67	35.91
	Acetamiprid	1289.21(1025.58–1772.77)	1.57(0.23)	0.08	3	0.99	350	2432.47	1.17
	Profenofos	413.47(251.26–1210.58)	1.34(0.27)	0.02	3	0.99	350	135.56	2.09
	Lambda-cyhalothrin	533.82(382.90–960.01)	1.63(0.27)	0.58	3	0.90	350	196.98	1.40

^a^N Total larvae in a bioassay including control.

^b^RR resistance ratio, LC_50_ of field population and Methox-SEL strains/LC_50_ of Susceptible strain.

^c^ RR resistance ratio, LC_50_ of Methox-SEL strain/LC_50_ of field population.

Methoxy-SEL was 3531.67-fold and 35.91-fold more resistant than susceptible and field strains, respectively (Table 1). Average response of selection to methoxyfenozide was 82% after 72 hours of exposure (Table 3).

### Cross-resistance pattern

Cross-resistance testing revealed that selection to methoxyfenozide didn’t increase resistance to any tested insecticides compared to field strain (Table 1). However, there was a slight change of 2.09-fold resistance to profenofos (95% FLs overlapping).

### Maternal effects and sex linkage

**Fig 3:**
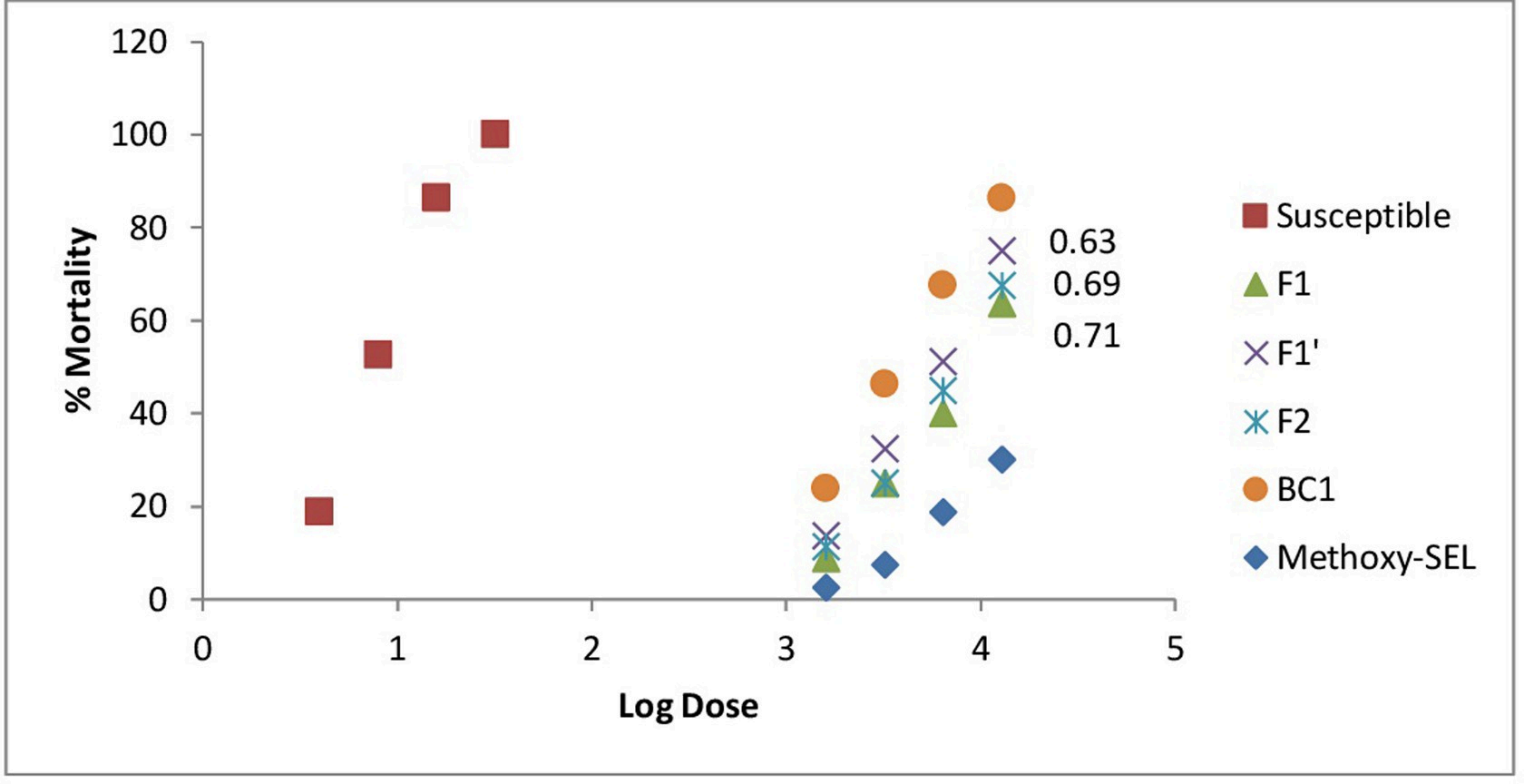
Percentage mortality of various crosses and strains with D_LC_ values.

Resistance in Methoxy-SEL was 3531.67-fold higher than susceptible strain. Resistance to methoxyfenozide dropped from 3531.67-fold to 1115.84-fold and 783.11-fold for F_1_ and F_1’_ reciprocal crosses, respectively, compared to Susceptible (Fig 3). LC_50_s of these reciprocal crosses were not significantly different suggesting an autosomal and no sex linkage was involved in resistance development.

#### Degree of dominance

**Fig 4:**
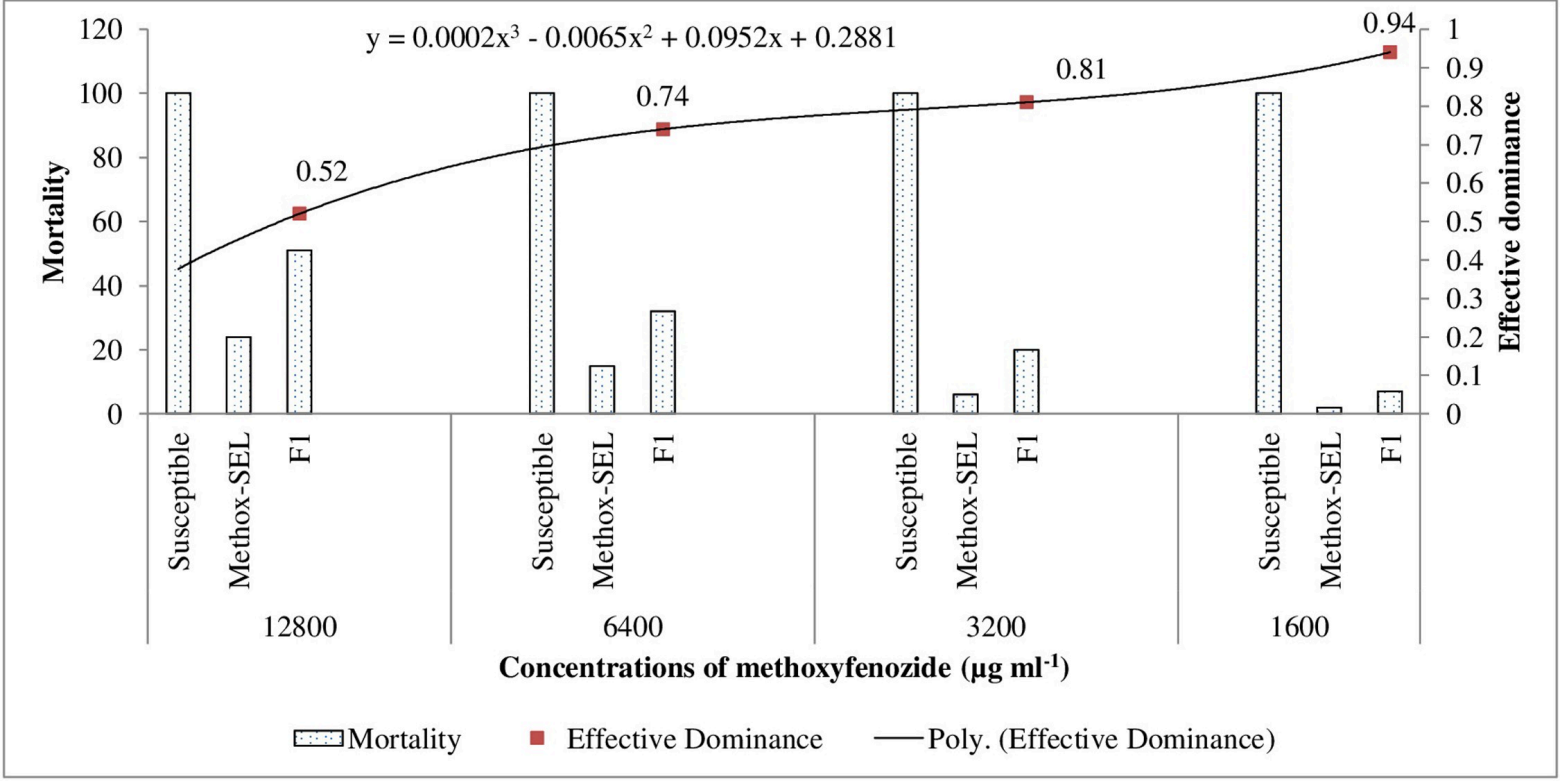
Effective dominance (D_ML_) of resistance in Methox-SEL strain of *Chrysoperla carnea*.

The D_LC_ values for F_1_, F_1’_ and F_2_ strains were 0.71, 0.63 and 0.69, respectively (Fig 3). These results suggest an incompletely dominant inheritance of methoxyfenozide resistance in *C*. *carnea*. The results for D_ML_ show that level of methoxyfenozide resistance dominance decreased when methoxyfenozide dose was increased from 1600 to 12800 μg ml^-1^. Resistance was likely completely dominant at lowest concentration (D_ML_ = 0.94) and incompletely dominant at highest concentration (D_ML_ = 0.52) tested (Fig 4).

#### Number of genes controlling resistance

**Fig 5:**
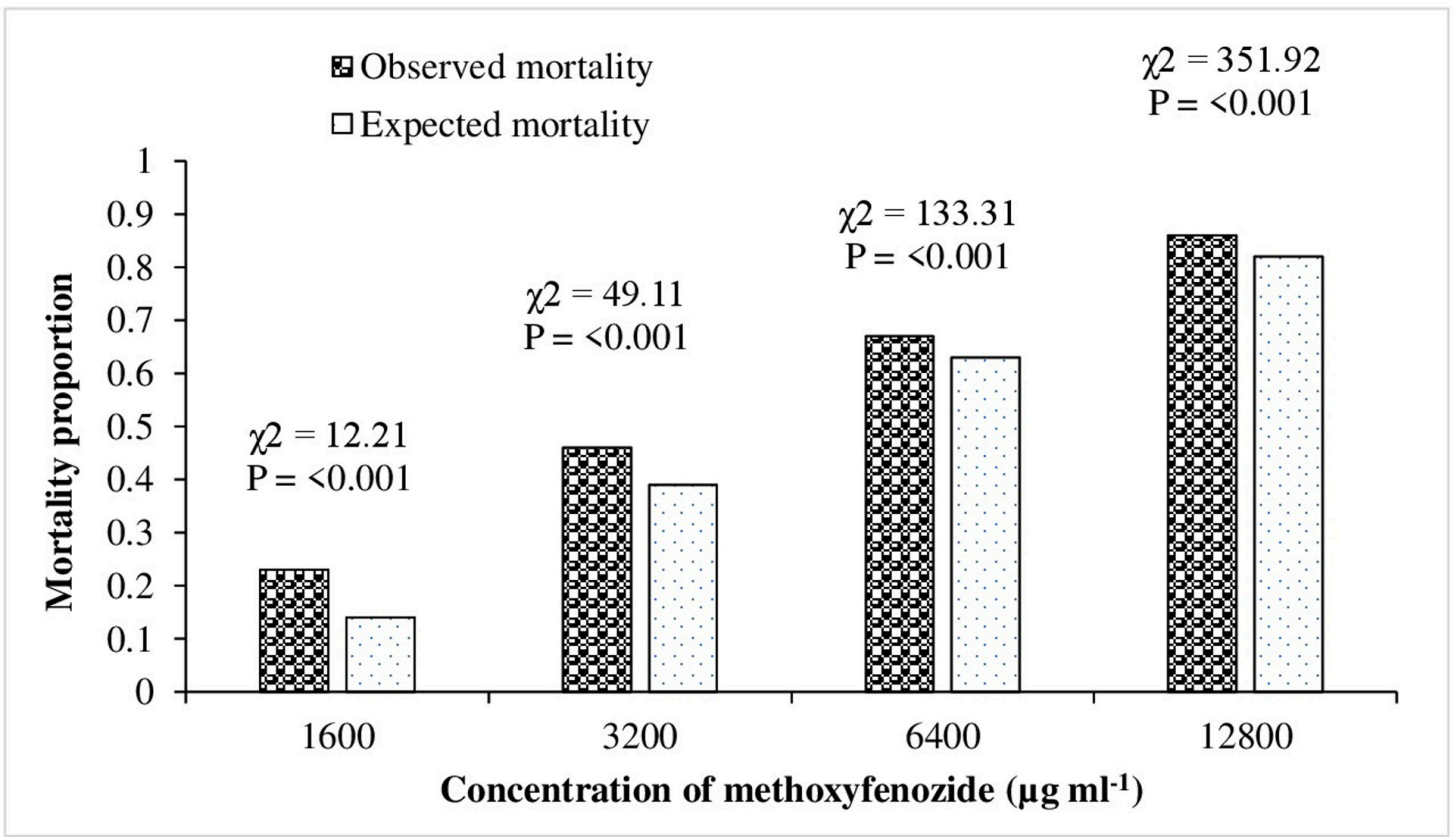
Direct test of monogenic inheritance of resistance to methoxyfenozide by comparing expected and observed mortality of the backcross (F1♀ x Susceptible ♂) of *Chrysoperla carnea*.

Monogenic test revealed that observed mortalities were significantly different (P<0.05) compared to expected results of mortality (Fig 5). These significant differences advocated that resistance to methoxyfenozide is polygenic.

### Realized heritability

The LC_50_ value increased from 731.51 to 26275.66 μg ml^-1^ in Methoxy-SEL strain after selecting 15 generations with methoxyfenozide. The realized heritability value of methoxyfenozide resistance was 0.62 (Table 2). The *h*^*2*^ value of methoxyfenozide resistance was high while expected number of generations required to gain a 10-fold increase in LC_50_ was only 10 (reciprocal of R: Table 2).

**Table 2:** Estimation of the realized heritability of resistance in Methox-SEL strain of *Chrysoperla carnea*.

	Estimation of mean selection response per generation				Estimation of mean selection differential per generation						
*N*	Insecticide	Initial log LC_50_ (μg ml^-1^)	Final log LC_50_ (μg ml^-1^)	Response to selection (*R*)	*P*	*I*	Initial slope	Final slope	*σp*	Selection differential (*S*)	*h* ^2^
15	Methoxyfenozide	2.86	4.42	0.10	82	0.31	2.13	1.56	0.54	0.17	0.62

*N* is the number of generations exposed with Methoxyfenozide.

*P* is the average survival% of green lacewing larvae throughout the selection.

*i* is the intensity of selection.

*σp* is the phenotypic variation.

### Synergism tests

**Fig 6:**
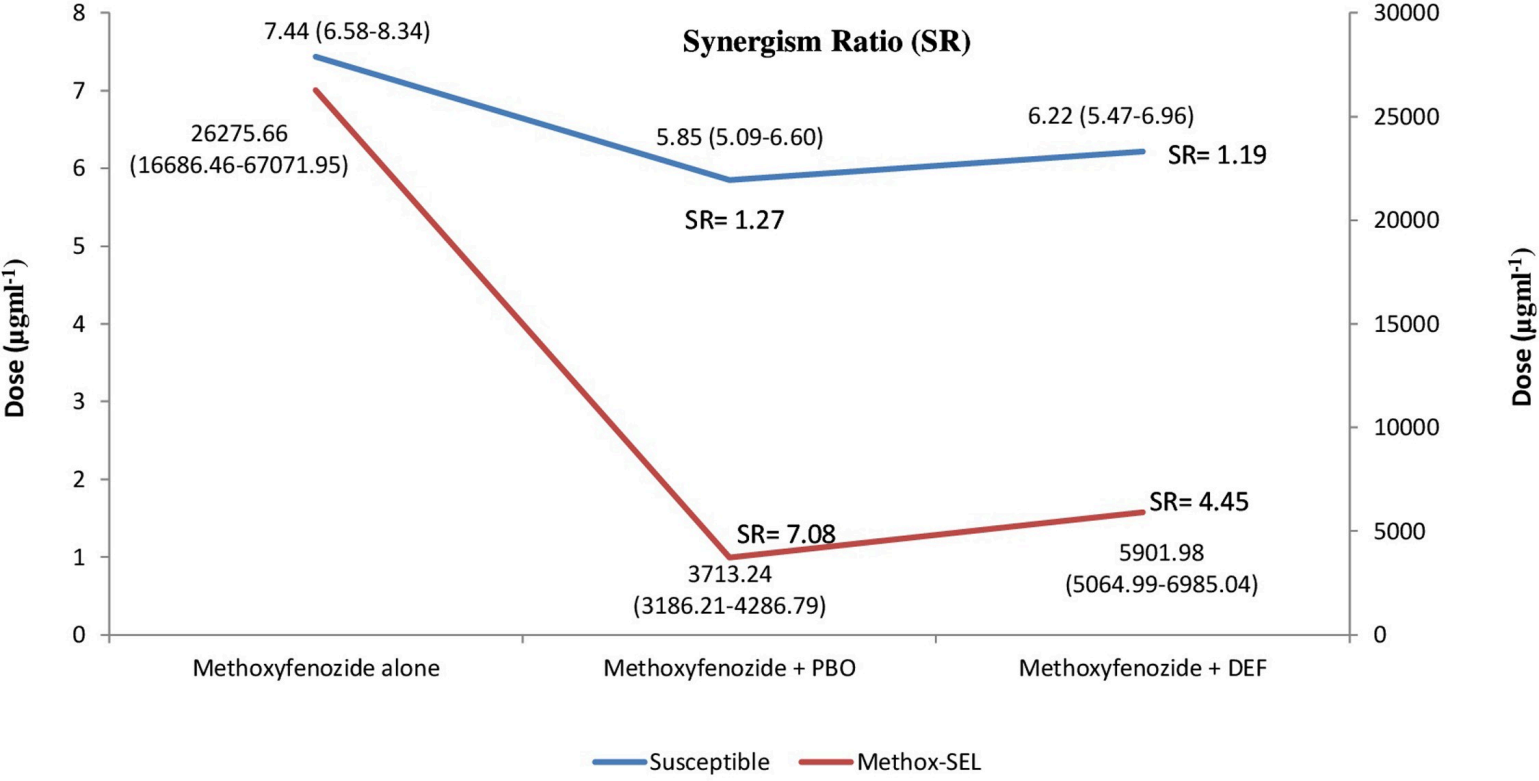
Toxicity of methoxyfenozide alone and with PBO or DEF to the susceptible and Methox-SEL *Chrysoperla carnea* strains.

Both PBO and DEF didn’t synergize the toxicity of methoxyfenozide against susceptible strain (95% FLs overlapping). However, toxicity of methoxyfenozide was significantly synergized by both PBO and DEF against Methoxy-SEL strain (95% FLs didn’t overlap). PBO and DEF decreased the LC_50_ values from 26275.66 to 3713.24 μg ml^-1^ and 26275.66 to 5901.98 μg ml^-1^, respectively, in Methoxy-SEL strain (Fig 6).

### Stability and decrease rate of resistance

Resistanceto all tested insecticides dropped significantly from G1 to G15. This suggested that resistance to methoxyfenozide and other sampled insecticides was unstable (95% FLs not overlapping). The decline rates for methoxyfenozide, acetamiprid, profenofos and lambda-cyhalothrin were -0.06, -0.08, -0.07, and -0.05, respectively. *C*. *carnea* would require at least 16, 13, 14 and 18 generations for a 10-fold decrease in resistance to methoxyfenozide, acetamiprid, profenofos and lambda-cyhalothrin, respectively (Table 3).

**Table 3:** Stability and decrease rate of resistance to methoxyfenozide and other tested insecticides in *Chrysoperla carnea*.

Strain	Insecticide	LC_50_ (95% FLs) μg ml^-1^	Fit of probit line				N^a^	RR^b^	DR	GR
			Slope (±SE)	χ2	df	*P*				
UNSEL (G15)	Methoxyfenozide	90.53(79.29–102.16)	3.28(0.32)	2.37	3	0.49	350	12.16	-0.06	16.53
	Acetamiprid	79.47(67.47–91.30)	2.83(0.30)	3.28	3	0.35	350	149.94	-0.08	13.14
	Profenofos	16.89(14.80–19.03)	3.41(0.34)	1.33	3	0.72	350	5.53	-0.07	14.06
	Lambda-cyhalothrin	57.38(51.09–64.21)	3.56(0.32)	2.88	3	0.41	350	21.17	-0.05	18.28

^a^ N = Total larvae used in bioassays including control.

^b^ RR resistance ratio, LC_50_ of UNSEL strain / LC_50_ of Susceptible strain.

## Discussion

Methoxyfenozide is a bio-rational insecticide and it acts as an ecdysone agonist. It is used to control numerous insect pests, especially pests from lepidoptera and diptera. After 15 generations of regular selection with methoxyfenozide, Methoxy-SEL strain of *C*. *carnea* developed 3531.67-fold resistance compared with susceptible strain.This showed immense potential of this natural enemy to develop resistance to this insecticide under selection pressure. There are numerous insecticide resistance reports for *C*. *carnea* [5, 7–10, 29, 41] indicating potential of this natural enemy in diverse field conditions.

Current resultsshowed that Methoxy-SEL developed no cross-resistance to acetamiprid (1.17-fold), lambda-cyhalothrin (1.40-fold) and profenofos (2.09-fold) compared to field strain (95% FLs overlapped). Previously, no cross-resistance to other insecticides in methoxyfenozide selected populations has been reported. For example, a population of *S*. *litura* (Fabricius) selected with methoxyfenozide showed no cross-resistance to profenofos, spinosad, fipronil, lufenuron, methoomyl or thiodicarb [24]. A methoxyfenozide-selected strain of *M*. *domestica* showed no cross-resistance to bifentrhin and spinosad but very low cross-resistance to chlorpyrifos and fipronil [25]. Obliquebanded leafroller, *Choristoneura rosaceana* (Harris) collected from various locations showed little cross-resistance between benzoylhydrazine, tebufenozide, methoxyfenozide and azinphosmethyl [42]. Lack of cross-resistance to different insecticides in Methoxy-SEL strain could be due to different resistance mechanisms [43, 44]. These results are in agreement with our previous findings of no, or little, cross-resistance exhibited by different insecticide-selected strains of *C*. *carnea* [7, 9, 10, 17].

Genetic crosses between insecticide-resistance and susceptible populations of natural enemies are a valuable tool to understand mode of inheritance, effective dominance, degree of dominance and number of genes supporting resistance development. Foreseeing how a natural enemy resistant to methoxyfenozide such as *C*. *carnea* may pass resistance to succeeding or susceptible populations and its possible impacts on insecticide resistance stability is only possible by studying genetics in laboratory. Reciprocal crosses F_1_, F_1’_ and F_2_ strains of *C*. *carnea* showed degree of dominance (D_LC_) values of 0.71, 0.63, and 0.69, respectively. These results indicated that LC_50_s were not significantly different in reciprocal crosses suggesting an autosomal mode of inheritance. These outcomes are similar to previous studies on inheritance of resistance to acetamiprid, pyriproxyfen and cyromazine [9, 11, 12]. These results are in agreement to a recent study concluding autosomal, and no sex linkage in methoxyfenozide resistance in *M*. *domestica* [26].

Studying effective dominance showed that methoxyfenozide resistance is incompletely dominant in Methoxy-SEL strain of *C*. *carnea*. Interestingly, resistance was likely completely dominant (D_ML_ = 0.94) at the lowest dose (1600 μg ml^-1^) tested. Changing concentration of any insecticide may affect dominance level [45]. Heritability of resistance increases due to increase in resistance dominance level, thus amplifying development of resistance. Dominance level for any particular factor is generally considered fixed but it may be affected by the genetic history and environmental circumstances [32]. Susceptible alleles may remain for a prolonged period even if resistance alleles show completely or incompletely dominant inheritance [46] and this situation supports interaction between dominant and recessive genes [47]. Current findings indicated that methoxyfenozide resistance increased rapidly due to selection pressure in *C*. *carnea* while selection levels, diverse environments, and population structures may cause dissimilar responses between field and laboratory populations. However, it also suggested that methoxyfenozide would not kill the heterozygotes easily as resistance to this insecticide is associated with dominant genes.

Monogenic resistance depended on chi-square (Goodness of fit test) and estimation of total number of generations showed that methoxyfenozide resistance is controlled by more than one gene. This suggests that *C*. *carnea* has polygenic resistance for methoxyfenozide. Polygenic resistance is common among field collected populations selected under laboratory conditions due to natural selection variations but monogenic resistance takes place in natural populations only [48]. However, Sayyed and Wright [49] reported that multiple genes controlling resistance could be evenly spread between natural and laboratory-selected populations. Our results concurred with previous studies reporting polygenic resistance to deltamethrin [17], buprofezin [10], acetamiprid [9], pyriproxyfen [11] and cyromazine [12] in *C*. *carnea*.

Realized heritability can be used effectively in assessing potential of increase in resistance and fate of an insecticide in laboratory-selected populations [35]. A high realized heritability value (0.62) suggests high genetic variation and quick increase in methoxyfenozide resistance in Methoxy-SEL strain of *C*. *carnea* after only 15 generations of selection. Furthermore, this strain would require only 10 generations (Reciprocal of R, Table 2) for a 10-fold increase in resistance. Even though laboratory conditions are not a true match of field conditions, the estimated *h*^*2*^ and predictable rate of methoxyfenozide resistance through laboratory selection have implications for biological control management. Therefore, this insecticide can be employed wisely in field.

Significant synergism by PBO and DEF on methoxyfenozide in Methoxy-SEL strain showed that cytochrome P450 monooxygenase and esterase might have a significant effect on detoxification of this insecticide. These results are in accordance with several reports of synergistic effects of PBO and DEF in methoxyfenozide-selected *S*.*exigua* [23], *S*. *litura* [24] and *M*. *domestica* [26]. Previously, Sayyed et al., [17], Mansoor et al., [7], Mansoor and Shad [10] and Mansoor and Shad [12] showed significant effect of PBO and DEF in synergizing the impact of deltamethrin, nitenpyram, buprofezin and cyromazine resistance in *C*. *carnea*, respectively.

Stability of insecticide resistance is a remarkable tool for result-oriented field utilization of natural enemies with idea of conservation. Natural enemies, such as *C*. *carnea* possessing insecticide resistance, which is stable, can be of benefit in IPM systems. This feature also ensures survival of non-targets especially when insecticide selection pressure is removed [7, 11]. Bioassays on UNSEL population of *C*. *carnea* concluded that resistance to methoxyfenozide and other insecticides was not stable. However, the decrease rate was minimal suggesting that this population will take at least 16 generations to show a 10-fold decrease in resistance. In contrast, the higher realized heritability (*h*^*2*^) and predictable rate of resistance development (reciprocal of R, Table 2) suggested that the same would need only 10 generations to acquire 10-fold resistance. This fact, accompanied with negligible decrease rates, also indicates the possibility of maintaining resistance for longer in field conditions if *C*. *carnea* receives periodic selection pressure of selected insecticides.

In conclusion, methoxyfenozide resistance in *C*. *carnea* is autosomal, incompletely dominant, controlled by multiple genes and settled by cytochrome P450-dependent monooxygenases and esterases. Although, hybrid individuals demonstrated incompletely dominant resistance, they also confer potential boost in resistance, suggesting that *C*. *carnea* has natural potential to acquire resistance to methoxyfenozide. Instable resistance in Methoxy-SEL and no cross-resistance to acetamiprid, profenofos and lambda-cyhalothrin with high to very high resistance in field populations suggested that these insecticides could be used in integration with *C*. *carnea* keeping in view the current resistance status of different pests.
